# St. Peter's Hospital

**Published:** 1902-04-05

**Authors:** 


### ST. PETER'S HOSPITAL.

The thirty-fourth annual meeting of St. Peter's Hospital for Stone and Urinary Diseases was held on Tuesday, the 26th ult., Mr. Fox presiding. As usual, the attendance of governors, though in fact rather above the average, was very limited, and this is the more to be wondered at since the statement on the affairs of the institution made by the chairman as well as those of the members of the surgical staff who form a large proportion of the attendance, are always lucid and interesting, as well as instructive with regard to the special work done by this hospital. The report showed that during 1901 490 in-patients were admitted, of whom 24 died, while 4 were discharged unrelieved. In the previous year 554 were treated, the decrease in 1901 being partly accounted for by the restriction of admissions during October, while one of the wards was being cleaned. The out-patients numbered 4,402, with 40,889 attendances. This was an increase on previous years; and the progress of exact surgery demanding every year greater scientific research and accuracy, additional help in the out-patient department was required. To meet this the committee appointed Mr. John Pardoe and Mr. J. W. Thomson Walker as surgical assistants. The attendance of 651 medical men on the practice showed the value attached to the work by those engaged in the study of the special branch of surgery to which the institution is devoted. The income for 1901 was £3,677, and the ordinary expenditure £3,981. Beyond that £271 was spent on building improvements; £100 of this was the cost of providing additional sleeping room for the servants, so as to arrange more adequate accommodation for the resident staff. The excess of expenditure over receipts for the year was £575, and to meet this the committee again reluctantly drew on the reserve capital, selling stock to the amount of £672. Towards the close of the year Lord Ardilaun notified that he wished to be relieved of the trusteeship he had held during the past 12 years, and Mr. S. J. Waring accepted the office in succession. In moving the adoption of the report, the Chairman congratulated the meeting on the fact that for the first time the hospital had been placed on the list of what was now King Edward's Fund.

